# The Preparation and Properties of a Shell Structure Ceramsite

**DOI:** 10.3390/ma13041009

**Published:** 2020-02-24

**Authors:** Wukui Zheng, Diyang He, Hui Li, Fei Wang, Yuxuan Yang, Jingjie Zhang

**Affiliations:** 1 College of Materials Science and Engineering, Xi’an University of Architecture and Technology, Xi’an 710055, China; zheng.wukui@xauat.edu.cn (W.Z.); he18149406571@163.com (D.H.); 15136458280@163.com (F.W.); yyx9202@163.com (Y.Y.); 15388618192@163.com (J.Z.); 2 Shanxi Ecological Cement & Concrete Engineering Technology Research Center, Xi’an 710055, China

**Keywords:** ceramsite, shell structure, lightweight, cylindrical compressive strength

## Abstract

In this paper, a shell structure ceramsite has been prepared and researched in order to attempt a new method of producing lightweight ceramsite. In the experiment, raw material was made into slurry and polypropylene balls were treated with the soak-and-pick process in the slurry to make the green body; later, the green body was dried and fired in the furnace to make the shell structure ceramsite. The result showed that the shell structure ceramsite has an appropriate cylindrical compressive strength (0.87 MPa) with a bulk density at a low level (0.375 × 103 kg/m^3^), which can be used for lightweight concrete preparation, and with its special structure, it can be used for many other purposes.

## 1. Introduction

Ceramsite is a kind of lightweight, porous, spherical or elliptical silicate ball with a certain strength, which is made of clay, shale, or solid waste. Because of its low density, high compressive strength, good impermeability, high porosity, heat preservation, and insulation, ceramsite is widely used as a building material [1,2,3], as a filter or in sound absorption materials [4,5,6], and as horticultural garden materials [7]. The application of ceramsite is also one of the important methods for solid waste recycling, and therefore the production and application of ceramsite (recycled aggregate) is becoming an important research direction.

As the process of ceramsite preparation has become relatively mature [1], researchers have focused on the functional and fine production of ceramsite [8]. Functionalized ceramsite includes various lightweight ceramsite, water absorbent ceramsite, and filtration ceramsite applications, such as performed by Zhao [9], who used volcanic ash and gold tailings to prepare a kind of lightweight high-strength ceramsite; Shao [10] used sludge and power plant fly ash to prepare lightweight ceramsite, which was able to meet the national standard requirements; and Liu [11] analyzed the relationship of ratio of the raw materials, firing temperature, and preheating temperature with the performance of ultralight ceramsite. Ma [12] discussed the pore-forming agent influence on lightweight ceramsite. For water absorbent and treatment, Jia [13] used lake sludge to prepare water-retaining ceramsite; Lu [14] studied the preparation and modification of porous lightweight ceramsite based on fly ash, the density of which is close to that of water and has good wear resistance. Jing [15] used tungsten slag to prepare ceramsite for copper-containing wastewater treatment. However, in the current research, whether the raw materials are sludge [16], metallurgical tailings [17], steel slag [18], or slag soil [19], the preparation methods are as simple as mixing and sintering. Although the research has achieved good results and played a positive role in the development of ceramsite, its porous structure always stays the same, which restricts its utilization. For example, lightweight ceramsite is difficult to further reduce due to its bulk density with its current structure.

In this paper, a shell structure ceramsite has been prepared and researched in order to obtain a lighter weight with an appropriate strength. With this special structure, it can also be applied in many different places, such as in lightweight concrete or soundproof material, which provides more application possibilities for ceramsite. The details are shown below.

## 2. Experiment

### 2.1. Shell Structure Ceramsite

**Figure 1:**
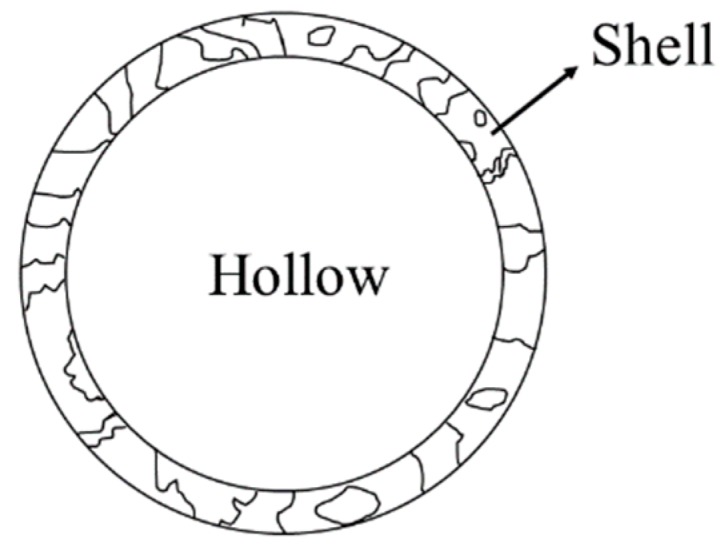
The diagram of the shell structure ceramsite.

In this paper, a shell structure ceramsite has been prepared and researched; its structure is a hollow ceramsite, as shown in Figure 1. This structure provides good mechanical properties from a compact shell, and greatly reduces density by being hollow; with this special structure, it can be applied in many different places compared with the traditional structure. In the preparation process of shell structure ceramsite, the burnable material is taken as the core, and the raw material of ceramsite is wrapped in the outer layer. At high temperature, the core material is burned and the shell material is sintered to form hollow ceramsite with a core-shell structure.

### 2.2. Raw Materials and Equipment

**Figure 2:**
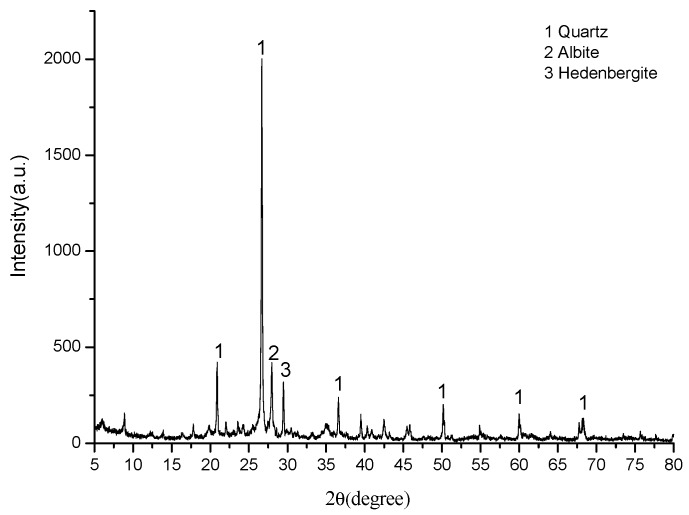
X-ray diffraction (XRD) analysis of clay used in the experiment.

Clay from Huxian in Xi’an city, China was used as the main material for making the shell structure ceramsite in the research. The chemical compositions are listed in Table 1, and the mineral phase composition is shown in Figure 2. 

The burnable material was a polypropylene ball with a diameter of 7–8 mm, and the thickener used in the experiment for the ceramsite green body formation was polyvinyl alcohol (Sigma Aldrich). The water used is distilled water. The furnace used in experiment was a chamber-type furnace from Carbolite Gero 1300-CWF (Carbolite-Gero, Hope Valley, UK).

### 2.3. Experimental Procedures and Method

**Figure 3:**
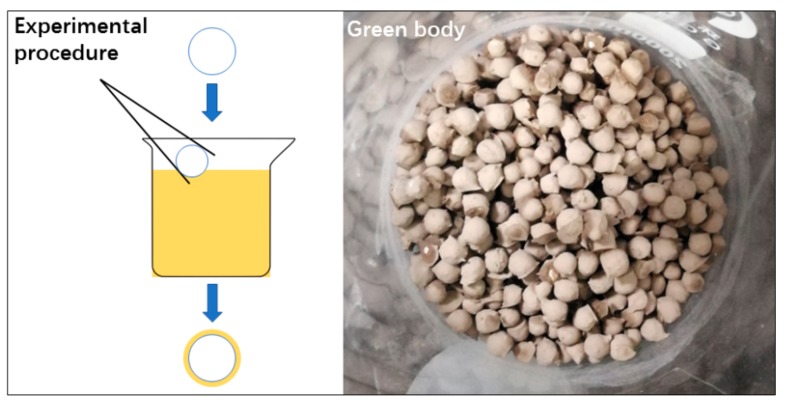
The experimental procedure of the shell structure ceramsite green body.

The clay was put into the oven for 24 h at 80 °C until its water content was lower than 0.5 wt.%. Then, we grinded the product in the ball mill for 3 h until all the material could pass through the 300 mesh. Later, the material was mixed with water (5 wt.% polyvinyl alcohol) to make a slurry, and the polypropylene ball was put into the slurry then picked out. With the help of the thickener, the slurry could easily stick to the surface of the polypropylene ball. The ball with the slurry (green body) was then dried at room temperature for 24 h. Finally, the green body was fired in a furnace to make shell structure ceramsite. The experimental procedure of making the green body is shown in Figure 3.

In the experiment, the influence of different temperatures for the shell structure ceramsite was researched. The temperature range in the experiment was from 1100 °C to 1200 °C. The heating rate was 10 °C per minute; the highest temperature was maintained for 30 min, and then the ceramsite was cooled with the furnace.

### 2.4. Testing Methods

The cylindrical compressive strength, water absorption, and bulk density were tested according to the National Standard of the People’s Republic of China “GB/T 17431.1-2010 Lightweight aggregates and its test methods-Part 1: lightweight aggregates”. 

X-ray fluorescence (XRF) analysis was utilized to determine the chemical composition of clay with all the samples passing through an 80 µm mesh using a Bruker S4 PIONEER XRF spectrometer (Bruker, Karlsruhe, Germany). The mineral phase analysis of clay and sintered products was carried out by D-MAX/2500 (Rigaku, Tokyo, Japan). Operating conditions were 40 kV and 40 mA using Cu–K radiation. The samples were scanned from 5° to 80°. The scanning speed was 1° per minute. The thermal analysis of clay was investigated by thermogravimetry (STA409PC, Netzsch, Gebrüder, Germany). In a typical measurement, 3 mg of sample (all passing through a 200-mesh screen) was heated in an Al_2_O_3_ crucible at a constant heating rate of 10 K/min in an air condition. The microscopic appearance of the samples was analyzed using a FEI Verios 460 scanning electron microscope (SEM) (Thermo Fisher Scientific, Waltham, USA).

## 3. Results and Discussion

**Figure 4:**
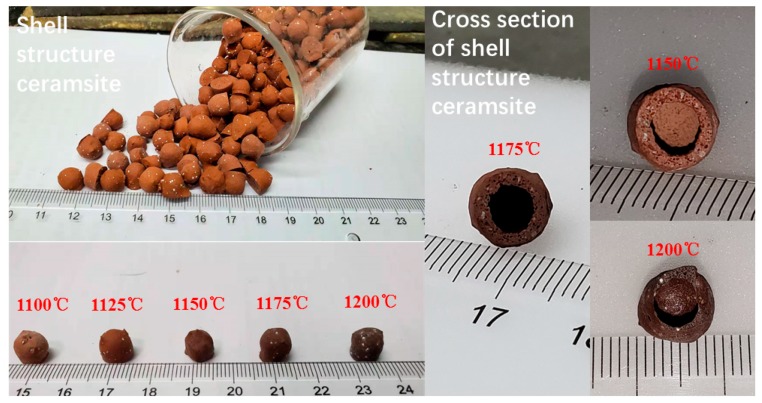
The appearance and cross section of shell structure ceramsite.

The appearance and cross section of shell structure ceramsite are shown in Figure 4. The ceramsite has a large hollow space inside with a ceramic of shell about 0.8–1.2 mm; with the increase of the temperature, a growth of the pore size is observed.

### 3.1. The Temperature Influence on Physical Properties of Shell Structure Ceramsite 

**Figure 5:**
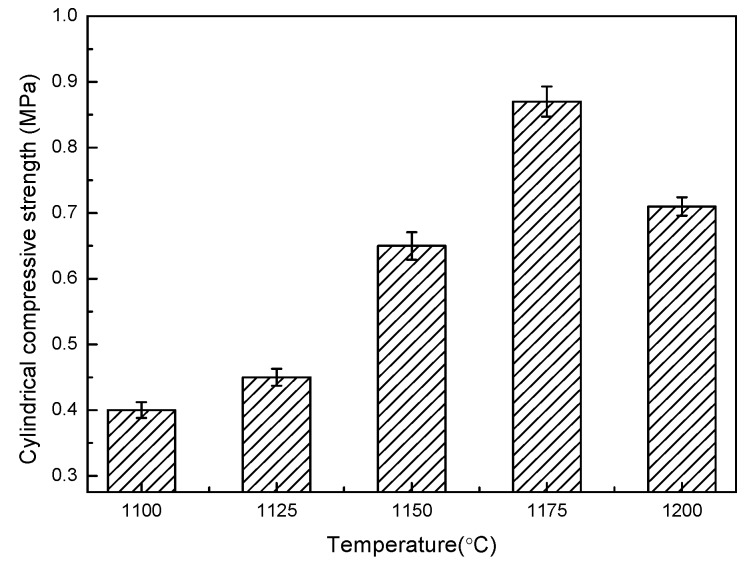
Cylindrical compressive strength of shell structure ceramsite.

**Figure 6:**
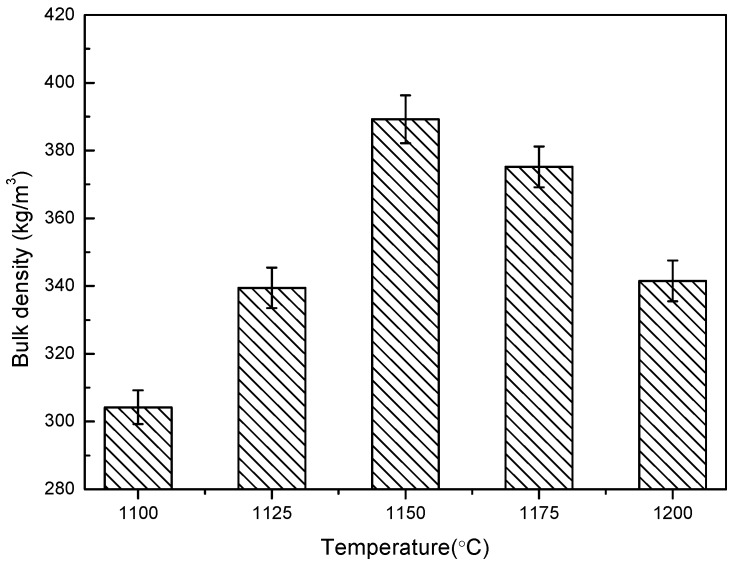
Bulk density of shell structure ceramsite.

**Figure 7:**
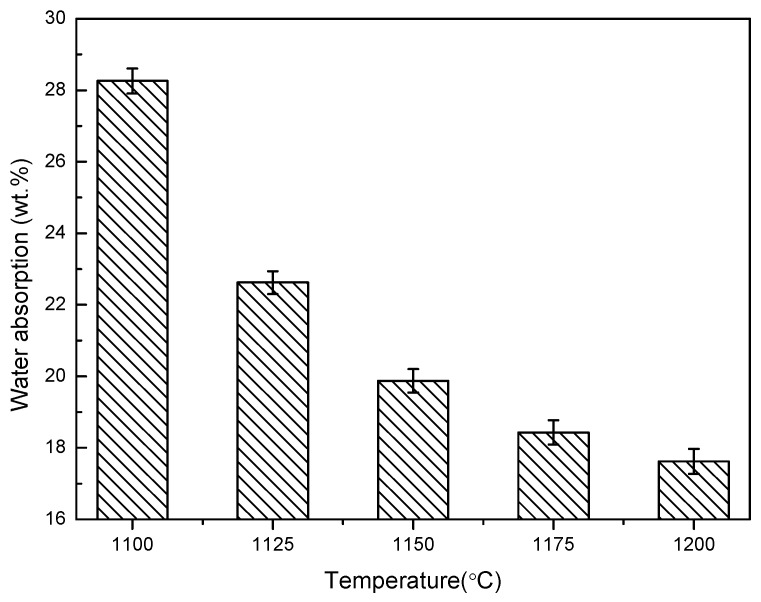
Water absorption of shell structure ceramsite.

The relationship between the physical properties of shell structure ceramsite, such as its cylindrical compressive strength, bulk density, and water absorption at different temperatures, is represented in Figure 5, Figure 6 and Figure 7. 

It can be seen from Figure 5 that, with the increase of sintering temperature, the strength increased, and the highest strength was 0.87 MPa at 1175 °C. After 1175 °C, the strength decreased. The lowest strength was 0.4 MPa at 1100 °C, and when the temperature was over 1200 °C, the samples would be overburnt; too much of the liquid phase was produced at high temperature, and the structure of the shell structure ceramsite was destroyed, resulting in the failure of preparation. This phenomenon in that the strength increases first and then decreases is different from the ordinary ceramsite. According to the research results of other researchers [20,21,22], the strength of ceramsite generally increases with the increase of temperature. However, some research results show a high point of strength, which is generally true in the study of bloating ceramsite. For example, in Hu’s research [23], a core-shell ceramsite also has the strength property of first increasing and then decreasing. This can be explained as follows: with the increase of temperature, the liquid phase gradually increases, the binding of the raw material particles is closer, and the strength of the ceramsite increases. With the further increase of temperature, a gas phase is produced in the raw materials, the ceramsite begin to bloat, voids increase, and strength decreases. 

It can be seen from Figure 6 that temperature has a great influence on the bulk density of shell structure ceramsite. With the increase of temperature, the bulk density gradually increased and started to decline after 1150 °C. The minimum bulk density was 304 kg/m^3^ at 1100 °C, and the maximum was 389 kg/m^3^ at 1150 °C. The difference between the shell structure ceramsite and ordinary ceramsite is that its shell structure has already prepared a large enough cavity to reduce the bulk density before the sintering process, but the bloating ceramsite reduces the bulk density due to the chemical reaction during sintering. This means that, with the increase of the liquid phase, there will be a certain degree of shrinkage due to the existence of an internal cavity, and the shrinkage will reduce the internal space and increase the bulk density. Therefore, the increase of the bulk density of shell structure ceramsite is more obvious than that of ordinary ceramsite. With the further increase of temperature, more gas was produced, which will increase the porosity and reduce the density of ceramsite.

It can be seen from Figure 7 that, with the increase of temperature, the water absorption gradually decreased, with the highest water absorption at 28.26% at 1100 °C, and the lowest at 17.62% at 1200 °C. As the shell structure has a hollow space inside, there was more space for the water. However, due to the air pressure, the internal cavity was not able to play the role of water absorption; thus, the cavity could not be filled with water, and there was a gap under the action of air pressure. Compared with the ceramsite with the same bulk density, the water absorption of the shell structure ceramsite was not high.

**Figure 8:**
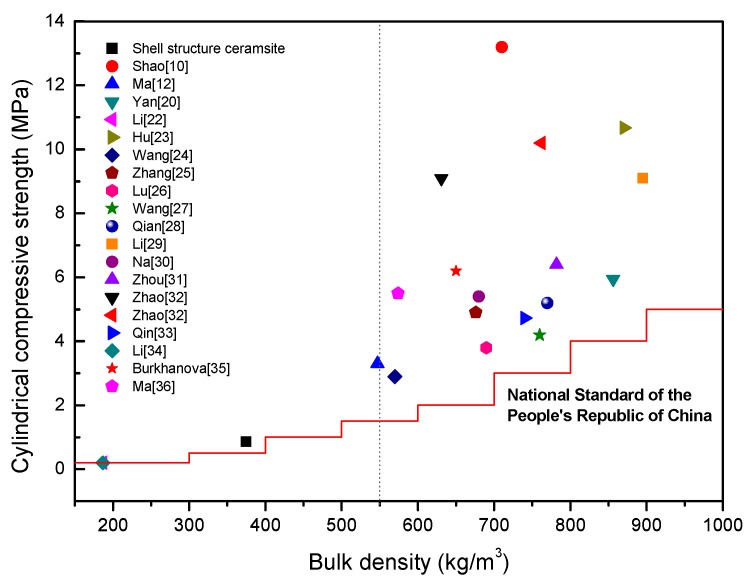
Comparison of ceramsite properties.

If the shell structure ceramsite’s cylindrical compressive strength and bulk density were put into the current research of lightweight ceramsite for comparison, as shown in Figure 8, it can be seen that the bulk density of lightweight ceramsite prepared by researchers is in the range of 550 kg/m^3^ to 900 kg/m^3^. High-strength ceramsite in this range is easy to produce, so many researchers have studied the application of various industrial solid wastes in lightweight high-strength ceramsite and have tried to prepare ceramsite with higher strength. Some researchers [10] could obtain ceramsite with a cylindrical compressive strength of 13.2 MPa and bulk density of 710 kg/m^3^. However, as shown in Figure 8 [10,12,20,22,23,24,25,26,27,28,29,30,31,32,33,34,35,36], there are few studies on ceramsite with a bulk density less than 550 kg/m^3^, because it is difficult to prepare ceramsite that meets the requirements by producing bloating ceramsite. In this case, the ceramsite with a shell structure can easily reduce the bulk density, and because the shell concentrates all the materials on the outside, it is beneficial to improve the strength. According to the current experimental results of shell structure ceramsite, although the results can meet Chinese national standards, the process still needs further optimization. The optimization should include the adjustment of raw materials, shell thickness, particle size, grading, and the balance between strength and density, in order to achieve better results to make up for the research gaps in the low-bulk-density range.

### 3.2. Micro Analysis of the Sintering Process

**Figure 9:**
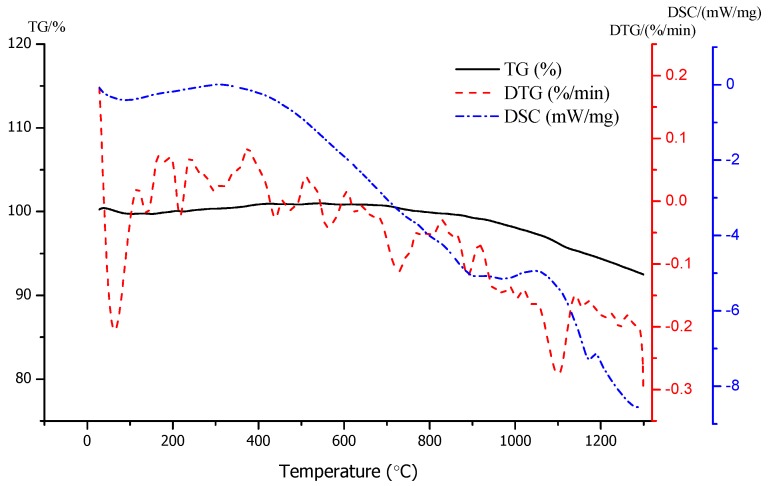
The thermogravimetric (TG) and differential scanning calorimetry (DSC) analysis of clay.

A thermogravimetric analysis of raw materials was carried out to study the sintering process, as shown in Figure 9. It can be seen from the figure that there was an obvious mass change and endothermic peak at 80–150 °C, which means a loss of water in the sample. With the increase of temperature, there was a weight loss process from 950 °C to 1200 °C, and the exothermic process started from about 950 °C, indicating that the sintering process started from about 950 °C. The weight loss rate increased gradually at 1050 °C, which indicates that gas generation started, and at around 1175 °C, there was an endothermic peak, which means the formation of new mineral phase. The thermogravimetric (TG) and differential scanning calorimetry (DSC) curves are a little bit earlier but consistent with the result of the macro experiment. The possible reason for this result is that the heat conduction process is not rapid enough during the ceramsite sintering.

**Figure 10:**
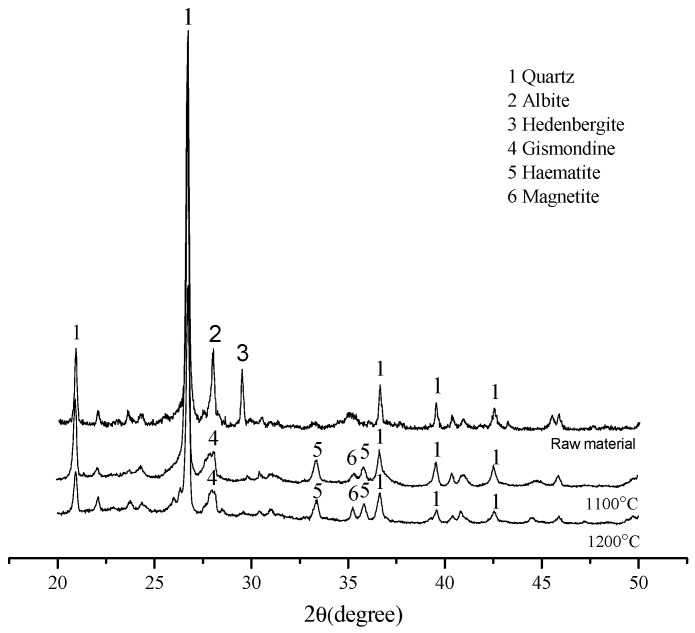
XRD analysis of shell structure ceramsite.

The XRD diagram of the raw material is compared with the sintered ceramsite at different temperatures, as shown in Figure 10. The main component in the raw material is silica, which also contains albite and hedenbergite. In Figure 10, it can be seen that, with the increase of temperature at 1100 °C, besides a large amount of silica, new mineral phases were formed, such as gismondine, haematite, and magnetite. With the further increase of temperature, due to the partial chemical reaction, the mineral phase formed was transformed from magnetite to haematite. This also caused a change in color and release of oxygen in the process. However, the reaction was not violent, and the peak in the figure did not change significantly, and so the shell structure of ceramsite was not damaged. This is also consistent with the results of macro experiments.

**Figure 11:**
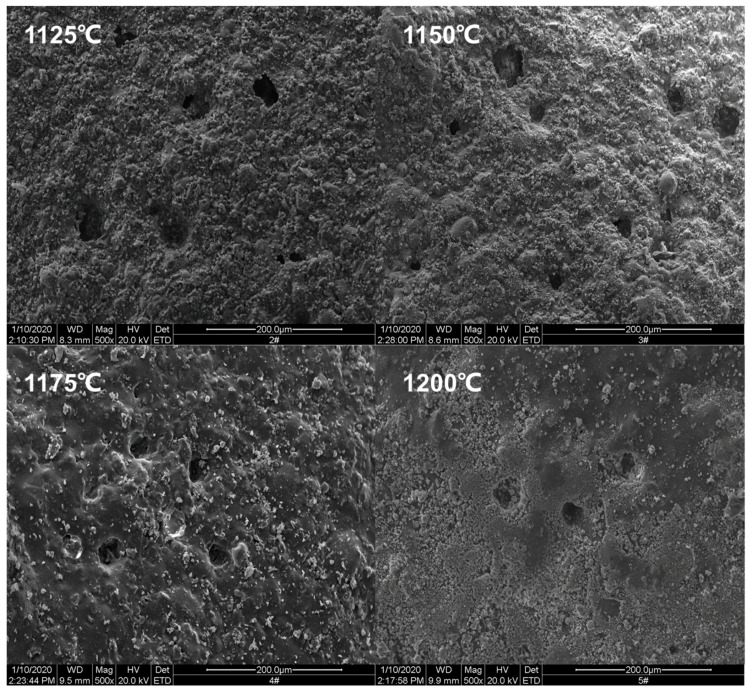
SEM analysis of ceramsite surface at different temperatures.

The SEM picture has been taken to analyze the ceramsite surface at different sintering temperatures, as shown in Figure 11. It can be seen from the figure that, when the temperature was 1100 °C, there were many particles on the surface and the particles sizes were large; no smooth surface formed due to the lack of the liquid phase. When the temperature was 1150 °C, the change of the surface was not obvious, but the surface particles changed from coarse particles to fine particles. At 1175 °C, the particles on the surface basically disappeared, there was enough liquid phase to make the surface smooth and dense, and the sintering reached the best state. At 1200 °C, it can be seen that the surface density reduced, and many tiny holes appeared due to the gas generation, thus causing a reduction of the cylindrical compressive strength and bulk density, which is consistent with the results of macro experiments.

From the above experimental analysis, it can be seen that for the shell structure type of ceramsite or ceramsite products, structural design should pay attention to the selection of raw materials that have no obvious reaction at a high temperature during sintering. The stability of the ceramsite structure can be ensured by the prejudgment of TG analysis or be checked by its chemical composition and mineral phase composition according to Riley’s [37] research results. It is important to determine whether the raw materials are suitable to be used in the preparation of ceramsite with a structural design such as a shell structure.

## 4. Conclusions

In this paper, the authors have presented a novel process of producing shell structure ceramsite, which is a simple process of producing lightweight ceramsite. The results showed the following:

(1) Shell structure ceramsite has an appropriate cylindrical compressive strength (0.87 MPa) with a bulk density at a low level (0.375 × 10^3^ kg/m^3^), which can be used for lightweight concrete preparation.

(2) Other than the common ceramsite, shell structure ceramsite or other sintered material uses structural design to reduce the density, which was already reduced from the beginning; the bloating process is harmful and will not reduce the density much. In this case, the material choice for shell structure ceramsite or other structured material should avoid bloating in the sintering process.

(3) Shell structure ceramsite has filled a gap in recent ceramsite research. Researchers have mainly studied the strength improvements of ceramsite, but the research on reducing the density and maintaining a certain strength is relatively sparse. The research on reducing the strength of ceramsite through hollow, sandwich, or other structural methods should also be paid attention to, so that the arrangement of ceramsite strength research should be focused not only on the high-strength area, but also on the ultra-light area.

However, there are still many problems in the preparation process. In the future, the production process has to be improved in order to be fit for mass production. Furthermore, the quality of the shell structure ceramsite has not reached its optimal quality, and more research has to be done to improve its quality. More research will also be done to test the other properties of the shell structure and try to apply it in more fields.

## Figures and Tables

**Table 1:** Chemical composition of clay (wt.%).

Component	SiO_2_	Al_2_O_3_	Fe_2_O_3_	CaO	K_2_O	MgO	Na_2_O	SO_3_	Others
Content (wt.%)	57.14	24.03	4.979	4.77	3.39	1.53	1.02	0.036	3.105
